# On the ***Lathrobium*** fauna of the Emei Shan, Sichuan, China (Coleoptera, Staphylinidae, Paederinae)

**DOI:** 10.3897/zookeys.277.4671

**Published:** 2013-03-15

**Authors:** Volker Assing, Zhong Peng, Mei-Jun Zhao

**Affiliations:** 1 Gabelsbergerstr. 2, D-30163 Hannover, Germany; 2 Department of Biology, College of Life and Environmental Sciences, Shanghai Normal University, Shanghai, 200234, P. R. China

**Keywords:** Coleoptera, Staphylinidae, taxonomy, *Lathrobium*, new species, description, key to species, species groups, endemism, Emei Shan, Sichuan, China

## Abstract

Six species of *Lathrobium* Gravenhorst, 1802 from the Emei Shan, Sichuan, are described and illustrated: *Lathrobium iunctum* Assing & Peng **sp. n.**, *Lathrobium coniunctum* Assing & Peng **sp. n.**, *Lathrobium conexum* Assing & Peng **sp. n.**, *Lathrobium ensigerum* Assing & Peng **sp. n.**, *Lathrobium hastatum* Assing & Peng **sp. n.**, and *Lathrobium bisinuatum* Assing & Peng **sp. n.** Based on their primary and secondary sexual characters, they represent two distinct lineages, each of them comprising three species. A key to the species recorded from the Emei Shan is provided.

## Introduction

According to a recent checklist, 90 species of the speciose paederine genus *Lathrobium* Gravenhorst, 1802 have been recorded from mainland China (Assing in press). With few exceptions, they are micropterous, endemic to particular mountains or mountain ranges, and inhabit the leaf litter layer of intermediate to high-altitude forest or shrub habitats. Nine species have been reported from Sichuan, one from the Gongga Shan, two from the Labahe Natural Reserve, one from the Daxue Shan, four from the environs of Songpan in northern Sichuan (Min Shan and adjacent mountains), and one from the border region with Shaanxi (Micang Shan) (Assing in press; Peng et al. 2012; Schülke 2002). Not a single species was previously known from the Emei Shan. The geographically closest localities from where *Lathrobium* species were described are the Gongga Shan (approximately 140 km) and the Labahe Natural Reserve (approximately 120 km).

**Figure 1.**
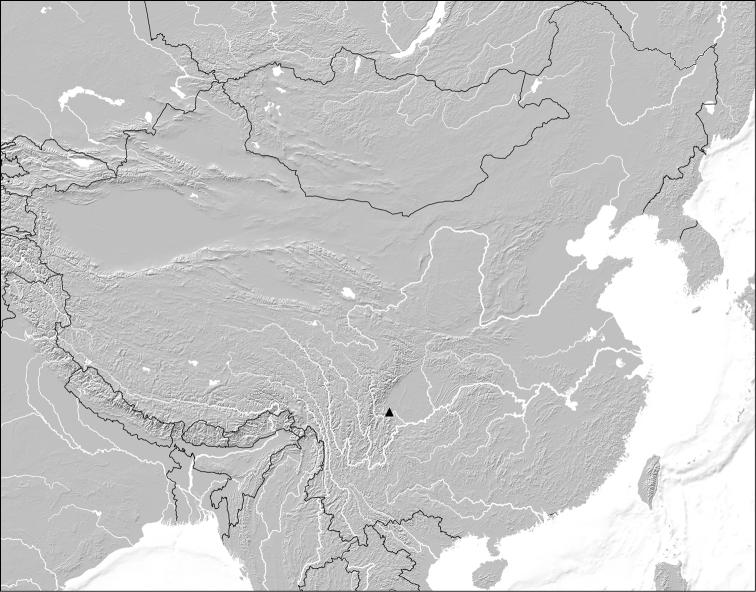
Geographic position of the Emei Shan in China.

Covering an area of 154 km^2^, the Emei Shan, one of the four sacred Buddhist mountains of China, is situated at the western rim of the Sichuan Basin and forms the southernmost part of the Qionglai range (Fig. 1), without being separated from adjacent mountains of this range by deep valleys. Geologically, the higher parts of the Emei Shan are dominated by igneous rock (basalt). The highest peak is the Wanfoding at 3,099 m. Below 1,800 m, the Emei Shan is subject to subtropical climate, at 1,800–2,200 m the climate is warm temperate, at 2,200–2,600 m it is medium temperate, and above 2,600 m it is cool temperate (Liu 1992). According to Li (1984) four vertical zones of forest vegetation can be distinguished: an evergreen broad-leaved forest zone at elevations below 1,900 m, a mixed evergreen and deciduous forest zone at 1,500-2,000 m, a mixed broad-leaved and coniferous forest zone at 2,000-2,500 m, and a cold-temperate coniferous forest zone at altitudes above 2,500 m. The Emei Shan is known to host numerous endemic plants and animals, among them at least three species of Paederinae, one of them in the genus *Nazeris* Fauvel, 1873 and two in *Rugilus* Leach, 1819 (Assing 2012a–b; Zheng 1992). One species of *Lobrathium* Mulsant & Rey, 1878 was described from the Emei Shan, too, but as the description is based on a single female, the species and its distribution are of doubtful status (Assing 2012c; Zheng 1988).

A study of recently collected *Lathrobium* material from the Emei Shan revealed a remarkable diversity. As many as six undescribed species were recognized; they represent the first records of the genus from this mountain.

## Material and methods

The material treated in this paper is deposited in the following public and private collections:

**CAS** Chinese Academy of Sciences, Beijing

**SNUC** Insect Collection of Shanghai Normal University, Shanghai

**cAss** private collection Volker Assing, Hannover

**cSme** private collection Aleš Smetana, Ottawa

The morphological studies were conducted using Stemi SV 11 (Zeiss Germany) and Olympus CX31 microscopes, and a Jenalab compound microscope (Carl Zeiss Jena). The images were prepared using Nikon Coolpix 995, Canon EOS 40D (with an MP-E 65 macrolens), and Canon G9 cameras. The map was created using MapCreator 2.0 (primap) software.

Body length was measured from the anterior margin of the mandibles (in resting position) to the abdominal apex, the length of the forebody from the anterior margin of the mandibles to the posterior margin of the elytra, head length from the anterior margin of the frons to the posterior margin of the head, elytral length at the suture from the apex of the scutellum to the posterior margin of the elytra, and the length of the aedeagus from the apex of the ventral process to the base of the aedeagal capsule. The “parameral” side (i.e., the side where the sperm duct enters) is referred to as the ventral, the opposite side as the dorsal aspect.

The labels are cited in the original spelling; different labels are separated by slashes.

## Results

Six new species are reported from the Emei Shan. All of them are micropterous (hind wings completely reduced) and most likely endemic to this mountain range.

### Species groups

The *Lathrobium* species of the Emei Shan undoubtedly belong to two distinct lineages, both of which are represented by three species.

One lineage is represented by the *Lathrobium iunctum* group and includes *Lathrobium iunctum*, *Lathrobium coniunctum*, and *Lathrobium conexum*. It is constituted by two evident synapomorphies, a male sternite VII with an obliquely asymmetric impression with numerous strongly modified short and stout black setae, as well as the asymmetric and fused ventral process and dorsal plate of the aedeagus. In addition, this group is characterized by dark coloration, moderately large size, a broad head (at least as long as broad), a broad pronotum (approximately 1.2 times as long as broad), the absence of a noticeable sexual dimorphism of the protarsomeres I-IV, a small basal portion of the aedeagus, a relatively short and posteriorly only weakly produced female sternite VIII, an anteriorly undivided and short median portion of the female tergite IX, and the long postero-lateral processes of the female tergite IX. This species group also includes *Lathrobium acutissimum* Peng et al., 2012 from the Labahe Natural Reserve in Sichuan and an undescribed species from the Qincheng Shan. For illustrations of *Lathrobium acutissimum* see Peng et al. (2012).

The second lineage is represented by the *Lathrobium ensigerum* group and comprises *Lathrobium ensigerum*, *Lathrobium hastatum*, and *Lathrobium bisinuatum*. This group is constituted particularly by the presence of a more or less distinctly sclerotized apical internal sclerite and by the shape of the dorsal plate of the aedeagus (apical portion strongly developed, distinctly sclerotized and long; basal portion reduced, very short). Additional characters characterizing this group are the oblong head, a moderately to very slender pronotum, small eyes, symmetric male sternites VII and VIII, a male sternite VIII with shallow posterior excision and with clusters of modified setae posteriorly, an aedeagus with a slender ventral process (at least in ventral view), a long and undivided median portion of the female sternite VIII (at least approximately as long as tergite X), and a posteriorly distinctly produced female sternite VIII. Although vastly different in size, *Lathrobium ensigerum* and *Lathrobium hastatum* apparently represent adelphotaxa, as is suggested by the synapomorphic presence of a long sclerotized spine in the internal sac of the aedeagus, by the similarly shaped dorsal plate of the aedeagus, by the derived shape of the male sternite VIII (presence of a posterior pair of impressions; posterior excision with median projection), as well as by the similarly slender pronotum.

### Natural history

The *Lathrobium* material from the Emei Shan was sifted from the leaf litter and soil beneath broad-leaved trees, bushes, bamboo, and rhododendron. One species was collected at an altitude of 1,100 m, the remaining species at altitudes of 1,700–2,500 m, one of them primarily at high elevations from approximately 2,500 up to about 3,000 m. The labels attached to the material suggest that up to four species may have been found syntopically.

## Descriptions

### Lathrobium iunctum

Assing & Peng sp. n.

urn:lsid:zoobank.org:act:3DCFC4CA-D35F-4B46-8B24-C456BE93C95B

http://species-id.net/wiki/Lathrobium_iunctum

Figs 2A 3 9

#### Type material.

Holotype ♂: ‘CHINA: Sichuan, Prov. Emeishan City, Mt. Emeishan, 29°33'N, 103°20'E, 23.vii.2012, alt. 2,000–2,300 m, Dai, Peng & Yin leg. / Holotypus ♂ *Lathrobium iunctum* sp. n., det. Assing & Peng 2012' (SNUC). Paratypes: 10♂♂, 13♀♀: ‘P. R. CHINA, Sichuan, Emei Shan, 29°33.6'N, 103°20.6'E, 27.vi.–5.vii.2009, 1800-2400 m, siftings 11-17, V. Grebennikov'; 1♂: ‘P. R. CHINA, Sichuan, Emei Shan, 29°32.932'N, 103°20.466'E, 2310 m, 01.vii.2009, sifting 14, V. Grebennikov'; 1♂, 3♀♀: ‘P. R. CHINA, Sichuan, EmeiShan, 29°32'56.0"N, 103°20'28.0"E, 2310 m, 20.vi.2010, sifting 38, V. Grebennikov'; 1♀: ‘P. R. CHINA, Sichuan, EmeiShan, 29°33'36.3"N, 103°20'38.0"E, 1947 m, 15.vi.2010, sifting 33, V. Grebennikov'; 10♂♂, 9♀♀: ‘CHINA Sichuan, Emei Shan, Leidongping, 2500 m, 18.VII.1996, 29°32N, 103°21E C65 / collected by A. Smetana, J. Farkač and P. Kabátek' (Paratypes in CAS, cSme, and cAss).

#### Etymology.

The specific epithet (Latin, adjective: connected, fused) alludes to the merged ventral process and dorsal plate of the aedeagus.

#### Description.

**Figure 2.**
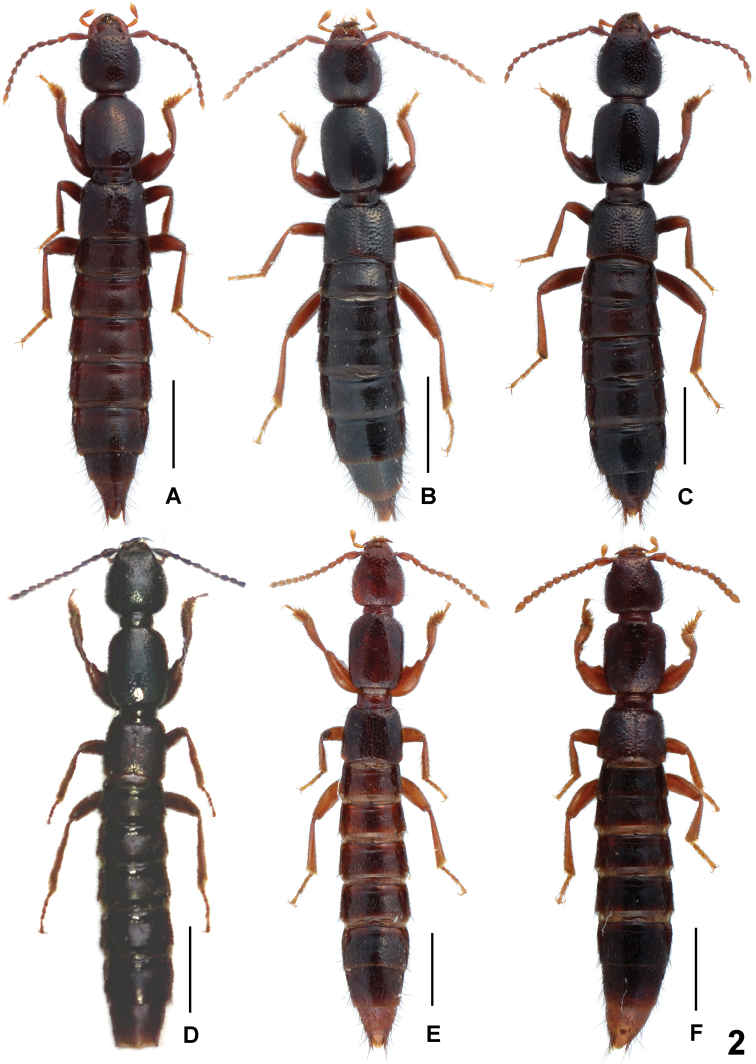
Habitus of *Lathrobium* spp., **A** *Lathrobium iunctum* **B** *Lathrobium coniunctum* **C** *Lathrobium conexum* **D** *Lathrobium ensigerum* **E** *Lathrobium hastatum* **F** *Lathrobium bisinuatum*. Scale bars: **A–C** 1.5 mm; **D** 2.0 mm; **E–F** 1.0 mm.

Species of moderately large and somewhat variable size, without sexual size dimorphism. Body length 8.0–9.5 mm; length of forebody 3.4–4.2 mm. Habitus as in Fig. 2A. Coloration: body blackish-brown to black, abdominal apex indistinctly paler; legs reddish-brown to dark-brown with pale-reddish tarsi; antennae reddish.

Head usually weakly transverse, 1.00–1.05 times as broad as long; punctation variable, moderately coarse to coarse and moderately dense to dense, sparser in median dorsal portion; interstices with fine but distinct microreticulation. Eyes weakly convex and rather large, approximately half the length of postocular region in dorsal view, or nearly so, and composed of numerous (> 50) ommatidia. Antenna 2.0–2.3 mm long.

Pronotum relatively broad, approximately 1.2 times as long as broad and 1.05–1.10 times as broad as head; punctation similar to that of head or somewhat finer; impunctate midline moderately broad; interstices without microsculpture.

Elytra short and broad, distinctly dilated posteriorly, approximately 0.55 times as long as pronotum, and at posterior margin approximately 1.6–1.7 times as broad (combined width) as long; punctation somewhat variable, usually shallow and moderately defined. Hind wings completely reduced. Protarsi without evident sexual dimorphism, moderately dilated in both sexes.

Abdomen with fine and rather dense punctation, that of tergite VII only slightly sparser than that of anterior tergites; interstices with fine microsculpture; posterior margin of tergite VII without palisade fringe; tergite VIII without sexual dimorphism, with truncate to weakly convex posterior margin.

**Figure 3.**
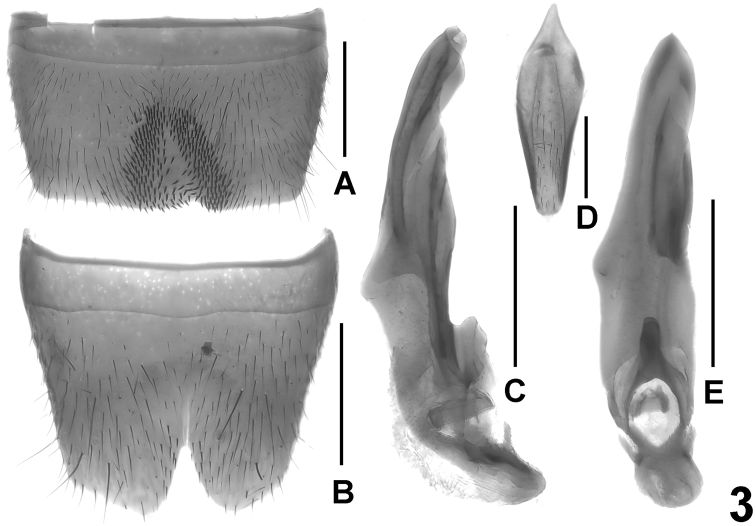
*Lathrobium iunctum*. **A** male sternite VII **B**﻿ male sternite VIII **C** aedeagus in lateral view **D** male sternite IX **E** aedeagus in ventral view. Scale bars: 0.5 mm.

Male. Sternites III-VI unmodified. Sternite VII strongly transverse, with asymmetric, somewhat oblique median impression posteriorly, this impression with pronounced and extensive cluster of numerous distinctly modified, short and stout black setae; posterior margin bisinuate or with shallow excision in asymmetric position (Fig. 3A). Sternite VIII transverse, pubescence unmodified; posterior excision almost symmetric, narrowly V-shaped and rather deep, its depth approximately 1/4–2/5 the length of sternite (Fig. 3B). Sternite IX as in Fig. 3D. Aedeagus (Figs 3C, E) approximately 1.5 mm long, slender, distinctly asymmetric, and with rather small basal portion; ventral process and dorsal plate fused; internal sac with small and weakly sclerotized basal sclerite.

**Figure 4.**
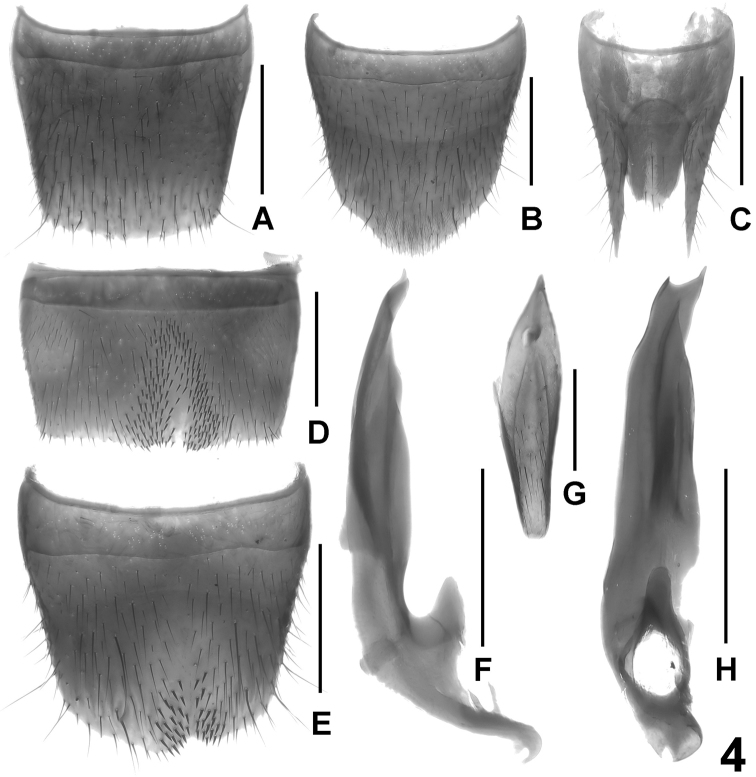
*Lathrobium coniunctum*. **A** female tergite VIII **B** female sternite VIII **C** female tergites IX–X. **D** male sternite VII **E** male sternite VIII **F** aedeagus in lateral view **G** male sternite IX **H** aedeagus in ventral view. Scale bars: 0.5 mm.

Female. Sternite VIII approximately as long as broad and with distinctly convex posterior margin, its shape similar to that of *Lathrobium coniunctum* (cf. Fig. 4B). Tergite IX undivided in the middle, with short median portion, and with long posterior processes; tergite X more than twice as long as tergite IX in the middle.

#### Comparative notes.

In external characters, *Lathrobium iunctum* is highly similar to the closely related *Lathrobium coniunctum* and *Lathrobium conexum* (see comparative notes in the following section), from which it is reliably distinguished only by the male sexual characters (shape and chaetotaxy of the male sternite VII; deep and narrow posterior excision of the male sternite VIII; shape of the aedeagus).

#### Distribution and natural history

**.** This species is most likely endemic to the Emei Shan, where the type specimens were sifted from leaf litter at elevations from approximately 1,800 to 2,500 m, together with *Lathrobium coniunctum*, *Lathrobium hastatum*, and *Lathrobium bisinuatum*. The locality where the holotype was collected is illustrated in Fig. 9.

### Lathrobium coniunctum

Assing & Peng sp. n.

urn:lsid:zoobank.org:act:15495984-54B5-4B11-9EF0-384631577BA6

http://species-id.net/wiki/Lathrobium_coniunctum

Figs 2B 4 10

#### Type material.

Holotype ♂: ‘CHINA: Sichuan Prov., Emeishan City, Mt. Emeishan, 29°33'N, 103°21'E, 21.vii.2012, alt. 1,700–1,900 m, Dai, Peng & Yin leg. / Holotypus ♂ *Lathrobium coniunctum* sp. n., det. Assing & Peng 2012'. Paratypes: 2♂♂: same label data as holotype; 2♀♀: same data, but ‘29°33'N, 103°20'E, 23.vii.2012, alt. 2,000–2,300 m' (SNUC); 1♂, 2♀♀: ‘P. R. CHINA, Sichuan, Emei Shan, 29°33.6'N, 103°20.6'E, 27.vi.–5.vii.2009, 1800–2400 m, siftings 11–17, V. Grebennikov'; 1♀: ‘P. R. CHINA, Sichuan, Emei Shan, 29°32.932'N, 103°20.466'E, 2310 m, 01.vii.2009, sifting 14, V. Grebennikov' (paratypes in SNUC, CAS, cSme, cAss).

#### Etymology.

The specific epithet (Latin, adjective: connected, fused) alludes to the merged ventral process and dorsal plate of the aedeagus and emphasizes the hypothesized close relationship of *Lathrobium coniunctum* to *Lathrobium iunctum* and the following species.

#### Description.

Body length 7.1–9.0 mm; length of forebody 3.4–3.8 mm. Habitus as in Fig. 2B. Legs reddish to reddish-brown. Other external characters as in *Lathrobium iunctum*.

Male. Sternites III-VI unmodified. Sternite VII distinctly transverse, with slightly asymmetric, somewhat oblique, and relatively extensive median impression, this impression with defined and extensive cluster of numerous distinctly modified, short and stout black setae; posterior margin bisinuate, with shallow excision in asymmetric position (Fig. 4D). Sternite VIII weakly transverse, with small and shallow, somewhat asymmetrically oblique median impression posteriorly, this impression with a cluster of distinctly modified, short and stout black setae on either side of middle; posterior excision shallow and in asymmetric position (Fig. 4E). Sternite IX as in Fig. 4G. Aedeagus (Figs 4F, H) approximately 1.4 mm long (from base of capsule to apex of dorsal plate), slender, distinctly asymmetric, and with small basal portion; ventral process and dorsal plate fused; dorsal plate apically obliquely bifid in ventral view; internal sac with small and weakly sclerotized basal sclerite.

Female. Sternite VIII approximately as long as broad and with distinctly convex posterior margin (Fig. 4B). Tergite IX undivided in the middle, with short median portion, and with long posterior processes; tergite X more than twice as long as tergite IX in the middle (Fig. 4C).

#### Comparative notes.

*Lathrobium coniunctum* is undoubtedly closely related to *Lathrobium iunctum*. This conclusion is supported by the similarly derived structure of the aedeagus (ventral process and dorsal plate fused, asymmetric, and slender; basal portion small; internal sac with small and weakly sclerotized basal sclerite); the similarly derived shape and chaetotaxy of the male sternite VII (median impression obliquely asymmetric and with defined, extensive cluster of distinctly modified setae), by the similar female secondary sexual characters (sternite VIII relatively short; tergite IX with short median portion and with long postero-lateral processes), as well as by the extremely similar external characters. Both species are best distinguished by the completely different shape and chaetotaxy of the male sternite VIII and by the differently shaped apex of the aedeagus.

#### Distribution and natural history.

Like *Lathrobium iunctum*, *Lathrobium coniunctum* is probably endemic to the Emei Shan, where the type specimens were sifted from leaf litter at elevations from approximately 1,700 to 2,310 m, partly together with *Lathrobium iunctum*. One of the collecting sites is illustrated in Fig. 10.

### Lathrobium conexum

Assing & Peng sp. n.

urn:lsid:zoobank.org:act:9A08D9F0-C476-4E8D-8872-1E25B3125646

http://species-id.net/wiki/Lathrobium_conexum

Figs 2C 5 11

#### Type material.

Holotype ♂: ‘CHINA: Sichuan Prov., Emeishan City, Mt. Emeishan, 29°33'N, 103°23'E, 27.vii.2012, alt. 1,100 m, Dai, Peng & Yin leg. / Holotypus ♂ *Lathrobium conexum* sp. n., det. Assing & Peng 2012' (SNUC). Paratypes: 2♂♂, 2♀♀: same data as holotype (SNUC).

#### Etymology.

The specific epithet (Latin, adjective: connected) refers to the merged ventral process and dorsal plate of the aedeagus and emphasizes the hypothesized close relationship of *Lathrobium conexum* to *Lathrobium iunctum* and *Lathrobium coniunctum*.

#### Description.

Body length 8.8–10.0 mm; length of forebody: 4.1–4.5 mm. Habitus as in Fig. 2C. Head noticeably transverse, 1.05–1.10 times as broad as long. Other external characters as in *Lathrobium iunctum*.

**Figure 5.**
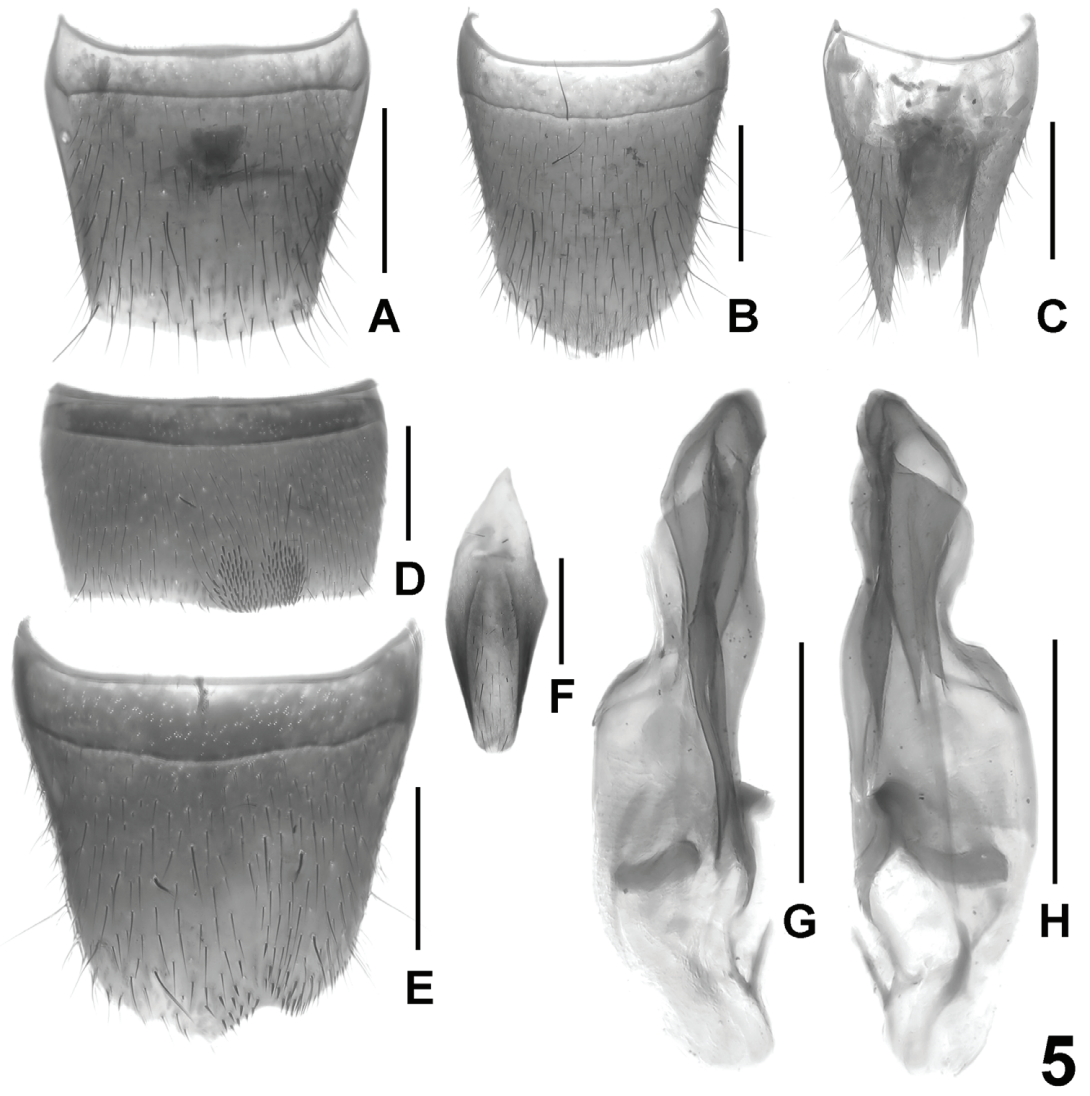
*Lathrobium conexum*. **A** female tergite VIII **B** female sternite VIII **C** female tergites IX–X**D** male sternite VII **E** male sternite VIII **F** male sternite IX **G** aedeagus in lateral view **H** aedeagus in ventral view. Scale bars: 0.5 mm.

Male. Sternite VII (Fig. 5D) distinctly transverse and with relatively small, subelliptic, and shallow posterior impression in asymmetric position, this impression with defined and extensive cluster of numerous distinctly modified, short and stout black setae; posterior margin weakly convex. Sternite VIII (Fig. 5E) weakly transverse, with small and shallow impression in asymmetric position posteriorly, this impression with few short black setae; posterior excision shallow and in distinctly asymmetric position. Sternite IX as in Fig. 5F. Aedeagus (Figs 5G, H) approximately 1.4 mm long (from base to apex of dorsal plate) and distinctly asymmetric; ventral process and dorsal plate fused; basal portion of aedeagus small; internal sac with weakly sclerotized basal sclerite.

Female. Sternite VIII (Fig. 5B) oblong, its posterior margin strongly convex. Tergite IX with short and undivided median portion and with moderately long postero-lateral proce sses; tergite X approximately 2.3 times as long as tergite IX in the middle (Fig. 5C).

#### Comparative notes.

As can be inferred from the similarly derived morphology of the aedeagus (ventral process and dorsal plate fused and distinctly asymmetric; small basal portion; internal sac with weakly sclerotized basal sclerite), the similar modifications of the male sternite VII (posterior impression in asymmetric position and with cluster of distinctly modified setae), the similar female secondary sexual characters, and the practically identical external characters, *Lathrobium conexum* is closely allied to *Lathrobium iunctum* and *Lathrobium coniunctum*. The similar modifications of the male sternite VIII would suggest that it may be most closely related to the latter.

#### Distribution and natural history.

This species is currently known only from the type locality. The specimens were collected by sifting leaf litter and humus from the floor of hardwood forest with *Kalopanax* at an altitude of 1,100 m (Fig. 11).

### Lathrobium ensigerum

Assing & Peng sp. n.

urn:lsid:zoobank.org:act:832FD0BE-F4F7-4FAA-A9C4-12346873B799

http://species-id.net/wiki/Lathrobium_ensigerum

Figs 2D 6

#### Type material.

Holotype ♂: ‘P. R. CHINA, Sichuan, EmeiShan, 29°32'57.2"N, 103°20'37.7"E, 16.vi.2010, 2289 m, sifting35, V. Grebennikov, Holotypus ♂ *Lathrobium ensigerum* sp. n., det. V. Assing 2012' (CAS). Paratype ♀: same data as holotype (cAss).

#### Etymology.

The specific epithet (Latin, adjective: carrying a sword) alludes to the shape of the ventral process of the aedeagus.

#### Description.

Large species; body length 11.0–13.0 mm; length of forebody 5.5–5.8 mm. Habitus as in Fig. 2D. Coloration: head and pronotum blackish-brown to black; elytra reddish-brown; abdomen brown to dark-brown, apex (posterior margin of segment VII; segments VIII-X) slightly paler; legs and antennae reddish to reddish-brown, antennomere I somewhat infuscate.

Head weakly oblong, approximately 1.05 times as long as broad; punctation not particularly coarse and moderately dense, sparser in median dorsal portion; interstices with fine but distinct microreticulation. Eyes weakly convex and rather small, less than one third or even only one fourth as long as postocular region in dorsal view. Antenna 3.2–3.3 mm long.

Pronotum slender, approximately 1.35–1.40 times as long as broad and 0.95 times as broad as head; punctation similar to that of head or somewhat finer; impunctate midline broader posteriorly than anteriorly; interstices without microsculpture.

Elytra short, not distinctly dilated posteriorly (i.e., lateral margins subparallel in dorsal view), approximately 0.50–0.55 times as long as pronotum; punctation shallow and dense. Hind wings completely reduced. Protarsi with weakly pronounced sexual dimorphism.

Abdomen with fine and rather dense punctation, that of tergite VII slightly sparser than that of anterior tergites; interstices with fine microsculpture; posterior margin of tergite VII without palisade fringe; tergite VIII without sexual dimorphism, in both sexes with weakly convex posterior margin.

**Figure 6.**
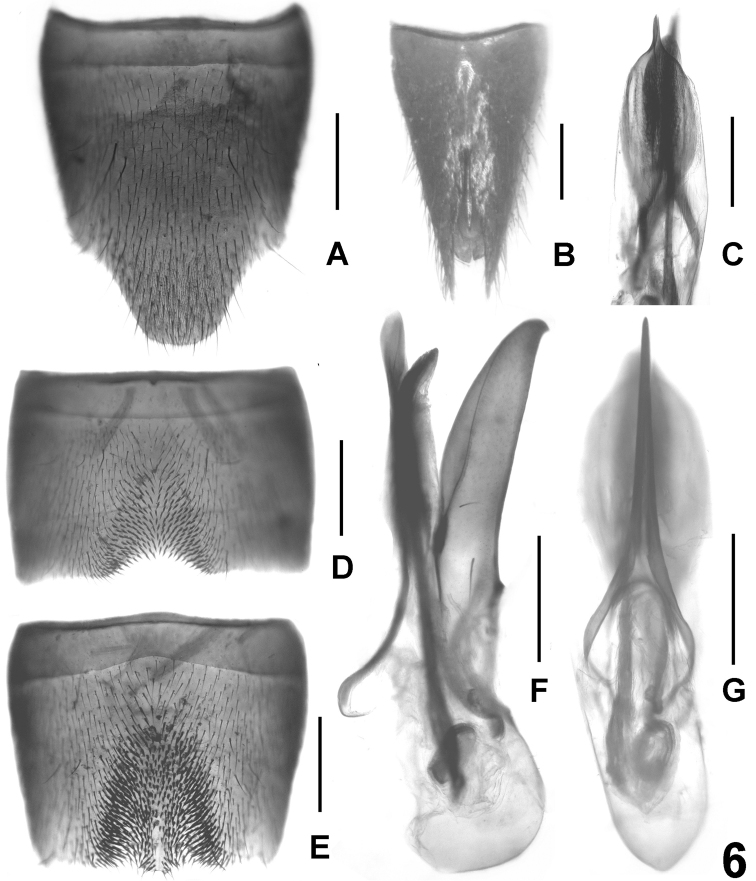
*Lathrobium ensigerum*.**A** female sternite VIII **B** female tergites IX-X C apical portion of aedeagus in dorsal view **D** male sternite VII **E** male sternite VIII **F** aedeagus in lateral view; **G** aedeagus in ventral view. Scale bars: 0.5 mm.

Male. Sternites III-VI unmodified. Sternite VII (Fig. 6D) strongly transverse, with median impression of triangular shape posteriorly, this impression with numerous distinctly modified, short and stout black setae; posterior margin distinctly concave in the middle. Sternite VIII (Fig. 6E) moderately transverse, with pair of posteriorly diverging impressions posteriorly, these impressions with numerous modified short black setae; posterior margin bisinuate, i.e., the shallow median excision with projection in the middle. Aedeagus (Figs 6C, F, G) 2.1 mm long and symmetric; ventral process blade-shaped, laterally compressed; dorsal plate with apical portion large, distinctly curved in lateral view, and apically acute in dorsal view; basal portion very short and thin; internal sac with long sclerotized spine and apically with additional, weakly sclerotized structure.

Female. Sternite VIII 1.7 mm long, distinctly oblong, posterior margin strongly produced in the middle (Fig. 6A). Tergite IX undivided in the middle, with long median portion, and with moderately long posterior processes; tergite X sharply keeled along the middle and approximately as long as tergite IX in the middle (Fig. 6B).

#### Comparative notes.

*Lathrobium ensigerum* is readily distinguished from all other species known from the Emei Shan by its much larger size alone. In addition, it is characterized by the slender pronotum, as well as the distinctive male and female sexual characters. For details regarding its phylogenetic affiliations see the comparative notes in the following section.

#### Distribution and natural history.

The type locality is situated in the Emei Shan. The specimens were sifted from leaf litter at an altitude of nearly 2,300 m.

### Lathrobium hastatum

Assing & Peng sp. n.

urn:lsid:zoobank.org:act:1AB56ED6-2A42-4768-B208-0B558633890A

http://species-id.net/wiki/Lathrobium_hastatum

Figs 2E 7 9

#### Type material.

Holotype ♂: ‘CHINA: Sichuan Prov., Emeishan City, Mt. Emeishan, 29°33'N, 103°20'E, 21.vii.2012, alt. 2,000–2,300 m, Dai, Peng & Yin leg. / Holotypus ♂ *Lathrobium hastatum* sp. n., det. Assing & Peng 2012' (SNUC). Paratypes: 6♂♂, 11♀♀: ‘P. R. CHINA, Sichuan, Emei Shan, 29°33.6'N, 103°20.6'E, 27.vi.–5.vii.2009, 1800-2400 m, siftings 11-17, V. Grebennikov'; 1♂, 2♀♀: ‘P. R. CHINA, Sichuan, EmeiShan, 29°32'56.0"N, 103°20'28.0"E, 2310 m, 20.vi.2010, sifting 38, V. Grebennikov'; 4♀♀: ‘P. R. CHINA, Sichuan, EmeiShan, 29°33'36.3"N, 103°20'38.0"E, 1947 m, 15.vi.2010, sifting 33, V. Grebennikov'; 1♀: ‘CHINA Sichuan, Emei Shan, Leidongping, 2500 m, 18.VII.1996, 29°32N, 103°21E C65 / collected by A. Smetana, J. Farkač and P. Kabátek' (Paratypes in CAS, SNUC, cSme, and cAss).

#### Etymology.

The specific epithet (Latin, adjective: armed with a spear) alludes to the presence of a long spine in the internal sac of the aedeagus.

#### Description.

Species of relatively small and somewhat variable size, without sexual size dimorphism. Body length 6.3–7.6 mm; length of forebody 2.9–3.3 mm. Habitus as in Fig. 2E. Coloration: body brown to blackish-brown, abdominal apex indistinctly paler; legs and antennae reddish.

Head weakly oblong, approximately 1.05 times as long as broad; punctation variable, relatively fine to moderately coarse and moderately sparse to moderately dense, sparser in median dorsal portion; interstices with fine but distinct microreticulation. Eyes weakly convex and small, approximately one fourth as long as postocular region in dorsal view and composed of approximately 20 weakly defined ommatidia. Antenna 1.6–1.8 mm long.

Pronotum slender, approximately 1.35 times as long as broad and approximately 1.05 times as broad as head; punctation similar to that of head or somewhat finer; impunctate midline moderately broad; interstices without microsculpture.

Elytra short, weakly dilated posteriorly, little more than 0.50 times as long as pronotum; punctation somewhat variable, usually shallow and moderately defined. Hind wings completely reduced. Protarsi with weakly pronounced sexual dimorphism.

Abdomen with fine and rather dense punctation, that of tergite VII slightly sparser than that of anterior tergites; interstices with fine microsculpture; posterior margin of tergite VII without palisade fringe; tergite VIII without sexual dimorphism, with truncate to weakly concave posterior margin.

**Figure 7.**
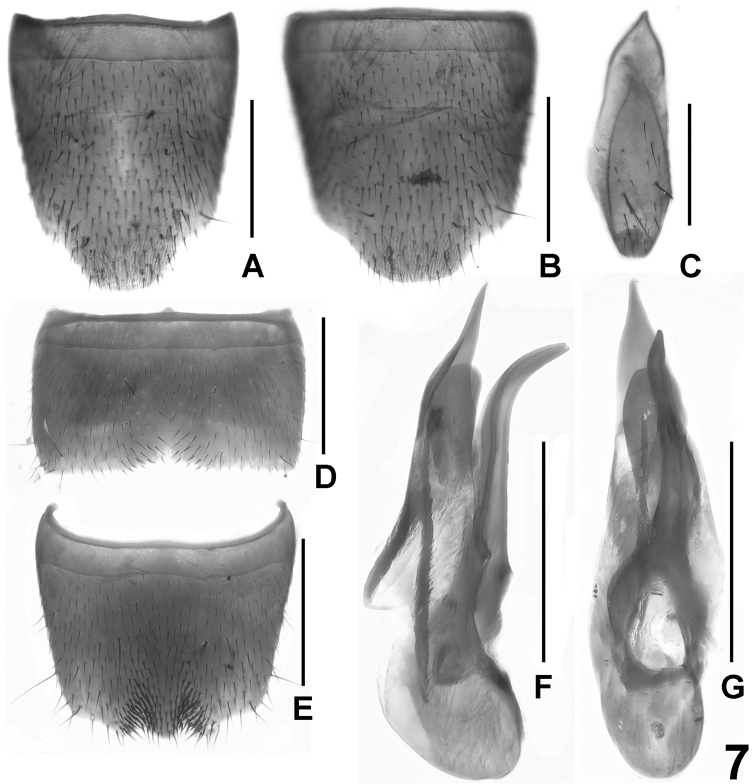
*Lathrobium hastatum*. **A**–**B** female sternite VIII **C** male sternite IX**D** male sternite VII **E** male sternite VIII **F** aedeagus in lateral view **G** aedeagus in ventral view. Scale bars: 0.5 mm.

Male. Sternites III-VI unmodified. Sternite VII strongly transverse, symmetric, with shallow median impression posteriorly, this impression with sparse and weakly modified dark setae (Fig. 7D). Sternite VIII moderately transverse, symmetric, with pair of small impressions posteriorly, these impressions with numerous distinctly modified, stout and dark setae; posterior margin bisinuate, i.e., the shallow posterior excision with median projection (Fig. 7E). Sternite IX as in Fig. 7C. Aedeagus (Figs 7F, G) approximately 1.1 mm long; ventral process subapically strongly curved and apically acute in lateral view; apical portion of dorsal plate large, long, apically very acute, and distinctly sclerotized; basal portion of dorsal plate very short; internal sac with long and straight sclerotized spine and with additional lamellate apical structure.

Female. Sternite VIII 0.9-1.0 mm long, weakly oblong, of slightly variable shape; posterior margin distinctly produced, middle weakly to distinctly convex (Figs 7A, B). Tergite IX undivided in the middle, with long median portion, and with relatively short postero-lateral processes; tergite X much shorter than tergite IX in the middle.

#### Comparative notes.

The similarly derived shape and chaetotaxy of the male sternite VIII (posteriorly with pair of impressions, these impressions with modified setae; posterior excision with median projection), the similarly derived morphology of the aedeagus (apical portion of dorsal plate large, apically acute, and distinctly sclerotized; basal portion of dorsal plate very short; internal sac with long sclerotized spine and apically with additional sclerite), the similar shape of the female sternite VIII, and the similarly slender pronotum suggest that, among the described species recorded from the Emei Shan, *Lathrobium hastatum* is most closely related to *Lathrobium ensigerum*. It is at once distinguished from this species by much smaller body size and by the sexual characters.

#### Distribution and natural history.

*Lathrobium hastatum* is probably endemic to the Emei Shan. The specimens were sifted from leaf litter at elevations between 1,800 and 2,500 m, together with *Lathrobium iunctum*, *Lathrobium coniunctum*, and/or *Lathrobium bisinuatum*. The locality where the holotype was collected is illustrated in Fig. 9. The ovaries of one of the dissected females contained a mature egg.

### Lathrobium bisinuatum

Assing & Peng sp. n.

urn:lsid:zoobank.org:act:009D2260-3423-4A80-89D7-652DA78EB784

http://species-id.net/wiki/Lathrobium_bisinuatum

Figs 2F 8 12

#### Type material.

Holotype ♂: ‘CHINA: Sichuan Prov., Emeishan City, Mt. Emeishan, 29°31'N, 103°20'E, 28.vii.2009, alt. 3,000 m, He & Tang leg. / Holotypus ♂ *Lathrobium bisinuatum* sp. n., det. Assing & Peng 2012' (SNUC). Paratypes: 5♀♀: same data as holotype; 1♂, 3♀♀: same data, but ‘17.vii.2009, alt. 3,000 m, Li-Zhen Li leg.'; 2♂♂, 3♀♀: same data, but ‘29°32'N, 103°20'E, 18.vii.2012, alt. 2,500–2,600 m, Dai, Peng & Yin leg.'; 9♂♂, 5♀♀: same data, but ‘19.vii.2012, alt. 2,800–3,000 m, Dai, Peng & Yin leg.'; 4♂♂, 1♀: same data, but ‘20.vii.2012, alt. 2,800–3,000 m, Dai, Peng & Yin leg.'; 1♂, 1♀: ‘P. R. CHINA, Sichuan, EmeiShan, 29°31'36.8"N, 103°19'52.1"E, 15.vi.2010, 2926 m, sifting 30, V. Grebennikov'; 4♀♀: ‘P. R. CHINA, Sichuan, Emei Shan, 29°33.6'N, 103°20.6'E, 27.vi.–5.vii.2009, 1800–2400 m, siftings 11-17, V. Grebennikov'; 3♀♀: ‘P. R. CHINA, Sichuan, EmeiShan, 29°30'46.5"N, 103°19'47.0"E, 14.vi.2010, 3035 m, sifting 28, V. Grebennikov'; 3♂♂, 3♀♀: ‘CHINA Sichuan, Emei Shan, Leidongping, 2500 m, 18.VII.1996, 29°32N, 103°21E C65 / collected by A. Smetana, J. Farkač and P. Kabátek' (Paratypes in CAS, SNUC, cSme, and cAss).

#### Etymology.

The specific epithet (Latin, adjective) alludes to the bisinuate posterior margin and the bisinuate dorsal plate (lateral view) of the aedeagus.

#### Description.

Small species without sexual size dimorphism. Body length 5.2–6.5 mm; length of forebody 2.6–2.8 mm. Habitus as in Fig. 2F. Coloration: body dark-brown to blackish-brown, abdominal apex indistinctly paler; legs and antennae reddish. Pronotum moderately slender, 1.21–1.26 times as long as broad. Posterior margin of tergite VIII truncate to weakly convex in both sexes. Other external characters as in *Lathrobium hastatum*.

**Figure 8.**
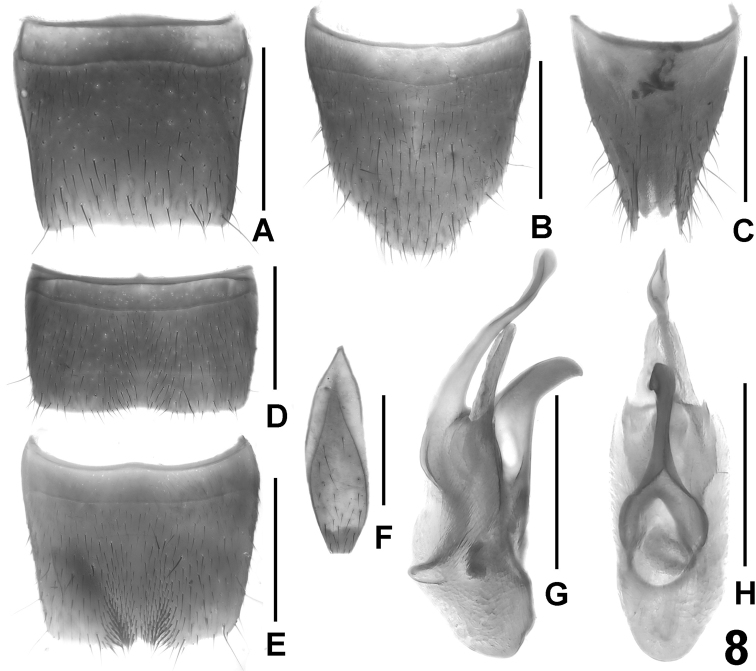
*Lathrobium bisinuatum*. **A** female tergite VIII **B** female sternite VIII **C** female tergites IX–X. **D** male sternite VII **E** male sternite VIII **F** male sternite IX **G** aedeagus in lateral view **H** aedeagus in ventral view. Scale bars: 0.5 mm.

Male. Sternites III-VI unmodified. Sternite VII strongly transverse, symmetric, with shallow median impression posteriorly, this impression with sparse and unmodified pubescence; posterior margin weakly and broadly concave (Fig. 8D). Sternite VIII moderately transverse, symmetric, shallowly impressed along the middle, this impression posteriorly with cluster of dense modified black setae on either side of middle; posterior excision very shallow, posterior margin on either side of this impression weakly concave (Fig. 8E). Sternite IX as in Fig. 8F. Aedeagus (Figs 8G, H) 0.9-1.0 mm long; ventral process laterally compressed, rather short, subapically curved, and apically acute; apical portion of dorsal plate of conspicuous shape, very long and slender, bisinuate in lateral view, and considerably projecting beyond apex of ventral process apically; basal portion of dorsal plate very short; internal sac with small and weakly sclerotized basal sclerite and with additional, semi-transparent apical sclerite.

Female. Sternite VIII 0.8–0.9 mm long, weakly oblong, posterior margin distinctly produced in the middle, apex of this projection truncate to weakly convex (Fig. 8B). Tergite IX undivided in the middle, with long median portion, and with relatively short postero-lateral processes; tergite X much shorter than tergite IX in the middle (Fig. 8C).

#### Comparative notes.

Among the *Lathrobium* species known from the Emei Shan, *Lathrobium bisinuatum* is most closely related to the species pair *Lathrobium ensigerum* + *Lathrobium hastatum*, a conclusion supported particularly by the structure of the aedeagus (presence of an apical internal sclerite; strongly developed apical portion and reduced basal portion of the dorsal plate) and additionally by the similar female secondary sexual characters (shape of sternite VIII; relative length and shapes of tergites IX and X), the somewhat similar shape and chaetotaxy of the male sternite VIII (posteriorly with shallow excision and with clusters of modified black setae), and the similar external characters. In external characters, *Lathrobium bisinuatum* is most similar to *Lathrobium hastatum*, from which it is distinguished by smaller size, darker average coloration, the less slender pronotum, as well as by the male sexual characters.

#### Distribution and natural history.

Like the other species described above, *Lathrobium bisinuatum* is probably endemic to the Emei Shan, where it was found primarily at high altitudes (2,500–3,000 m). In one locality at 2,500 m, *Lathrobium bisinuatum* was found together with *Lathrobium iunctum* and *Lathrobium hastatum*. Four females were collected somewhere between 1,800–2,400 m; they have the same label data as specimens of *Lathrobium iunctum*, *Lathrobium coniunctum*, and/or *Lathrobium hastatum*, suggesting that they were collected syntopically. Some of the specimens were sifted from rhododendron litter and humus in a rhododendron forest on a west slope near the mountain summit at an altitude of 2,800–3,035 m (Fig. 12).

**Figures 9–12.**
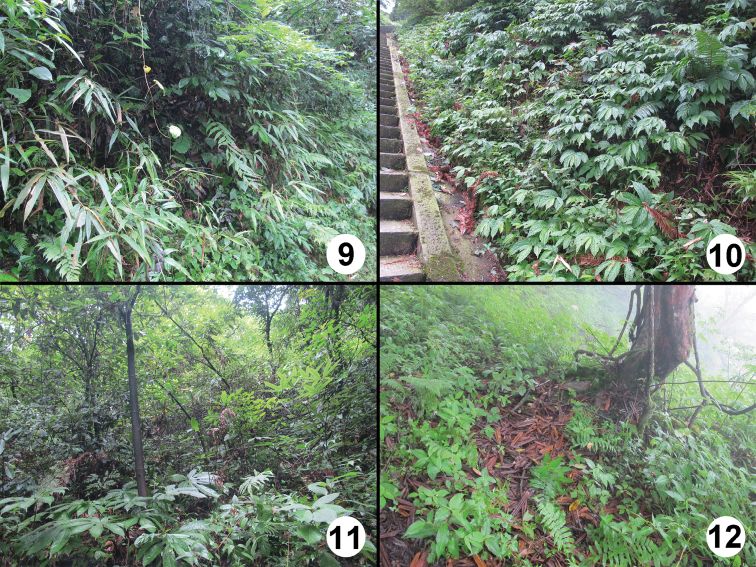
Collecting sites on the Emei Shan. **9** type localities of *Lathrobium iunctum* and *Lathrobium hastatum* (2,000–2,300 m) **10** site where *Lathrobium coniunctum* was collected (1,700–1,900 m) **11** type locality of *Lathrobium conexum* (1,100 m) **12** site where *Lathrobium bisinuatum* was collected (2,800–3,000 m).

### Key to the *Lathrobium* species of the Emei Shan

**Table:** 

1	Large species; body length > 10 mm; length of forebody > 5 mm. Head slightly broader than pronotum (Fig. 2D). ♂: aedeagus 2.1 mm long, with sword-shaped ventral process (Figs 6C, F, G); sternites VII and VIII of distinctive shapes and chaetotaxy (Figs 6D, E). ♀: tergite X sharply keeled along the middle, approximately as long as tergite IX in the middle (Fig. 6B)	*Lathrobium ensigerum*
–	Distinctly smaller species; body length < 10 mm; length of forebody < 4.5 mm. Head slightly narrower than pronotum. ♂: aedeagus much smaller and with ventral process of different shape; sternites VII and VIII of different shape and chaetotaxy. ♀: tergite X not keeled, either distinctly shorter or distinctly longer than tergite IX in the middle	2
2	Larger species; body length > 7.7 mm; length of forebody > 3.3 mm. Head as broad as long or weakly transverse. Eyes larger, approximately half as long as postocular region in dorsal view, or nearly so, and composed of > 50 ommatidia. Pronotum broad, approximately 1.2 times as long as broad. Elytra broad and distinctly dilated posteriorly. ♂: sternite VII with median impression of obliquely asymmetric shape, this impression with extensive cluster of short and stout black setae; aedeagus asymmetric and with small basal portion, ventral process and dorsal plate fused. ♀: sternite VIII weakly oblong at most, posterior margin convex, not strongly produced in the middle; tergite IX with short median portion and long postero-lateral processes; tergite X much longer than tergite IX in the middle	3
–	Smaller species; body length < 7.6 mm; length of forebody < 3.4 mm. Head weakly oblong. Eyes smaller, less than one third as long as postocular region in dorsal view, and composed of approximately 20 ommatidia. Pronotum more slender, > 1.2 times as long as broad. Elytra only weakly dilated posteriorly. ♂: sternite VII with symmetric impression, this impression with sparse unmodified or weakly modified setae; aedeagus symmetric or weakly asymmetric and with large basal portion, ventral process and dorsal plate not fused. ♀: sternite VIII distinctly oblong, posterior margin distinctly produced in the middle; tergite IX with long median portion and relatively short postero-lateral processes; tergite X much shorter than tergite IX in the middle	5
3	♂: sternite VIII with deep and symmetric posterior excision, pubescence unmodified (Fig. 3B); sternite VII as in Fig. 3A; aedeagus as in Figs 3C, E	*Lathrobium iunctum*
–	♂: sternite VIII with small and shallow posterior excision in asymmetric position, posteriorly with modified setae; aedeagus of different shape	4
4	♂: sternite VII with weakly convex posterior margin and with less extensive impression (Fig. 5D); sternite VIII with broader posterior excision and with fewer, weakly modified setae posteriorly (Fig. 5E); sternite IX less oblong (Fig. 5F); aedeagus stouter, dorsal plate not bifid apically in ventral view (Figs 5G, H). ♀: sternite VIII weakly oblong (Fig. 5B)	*Lathrobium conexum*
–	♂: sternite VII with shallow posterior excision and with more extensive impression (Fig. 4D); sternite VIII with smaller posterior excision and with more numerous and more distinctly modified setae posteriorly (Fig. 4E); sternite IX more slender (Fig. 4G); aedeagus more slender, dorsal plate bifid apically in ventral view (Figs 4F, H). ♀: sternite VIII approximately as long as broad (Fig. 4B)	*Lathrobium coniunctum*
5	Body larger; length of forebody 2.9–3.3 mm. Pronotum more slender, approximately 1.35 times as long as broad. ♂: sternite VII with weakly modified setae in posterior impression, posterior margin more distinctly concave in the middle (Fig. 7D); sternite VIII with median projection in the middle of the posterior excision; aedeagus approximately 1.1 mm long, with longer and more slender ventral process in lateral view, with broad and shorter dorsal plate, and with long sclerotized spine in internal sac (Figs 7F, G)	*Lathrobium hastatum*
–	Body smaller; length of forebody 2.6–2.8 mm. Pronotum less slender < 1.30 times as long as broad. ♂: sternite VII with unmodified pubescence in posterior impression and with weakly concave posterior margin (Fig. 8D); posterior margin of sternite VIII with small posterior excision, this excision without median projection (Fig. 8E); aedeagus 0.9–1.0 mm long with shorter and broader ventral process in lateral view, with conspicuously long, slender, and bisinuate (lateral view) dorsal plate, internal sac only with small basal sclerite in internal sac (Figs 8G, H)	*Lathrobium bisinuatum*

## Supplementary Material

XML Treatment for Lathrobium iunctum

XML Treatment for Lathrobium coniunctum

XML Treatment for Lathrobium conexum

XML Treatment for Lathrobium ensigerum

XML Treatment for Lathrobium hastatum

XML Treatment for Lathrobium bisinuatum
